# Closer in hard times? The drivers of European solidarity in ‘normal’ and ‘crisis’ times

**DOI:** 10.1057/s41295-023-00332-w

**Published:** 2023-02-07

**Authors:** Anna Kyriazi, Alessandro Pellegata, Stefano Ronchi

**Affiliations:** Department of Social and Political Sciences, Università degli Studi di Milano, Via Conservatorio 7, 20122 Milan, Italy

**Keywords:** European Union, Covid-19 crisis, Identity, Public opinion, Solidarity, Utility

## Abstract

This article examines the trends and differences in predictors of public support for European Union (EU) fiscal solidarity using two individual surveys conducted in 2019 and 2020, before and during the first wave of the Covid-19 pandemic, in six Western European countries. We focus on individual self-interest and European/national identification as the two major determinants of public preference formation. Empirical analyses show that, while the average level of public support for European fiscal solidarity did not change from 2019 to 2020, the negative associations between exclusive national identification and economic vulnerability, on the one hand, and EU fiscal solidarity on the other were weakened. Among both, the identitarian source retained substantive (although reduced) relevance in 2020, while utility did not. Country-level analyses reveal a more complex picture, but the overall pattern holds across the member states included in our sample. We argue that the reduced explanatory power of these typical heuristics that individuals use to shape their attitudes towards European solidarity is connected to the nature of the pandemic as an exogenous ‘common crisis’, affecting all member states in a supposedly symmetric manner, at least in the first phase, and inducing interdependencies among them.

**Supplementary Information:**

The online version contains supplementary material available at 10.1057/s41295-023-00332-w.

## Introduction

The European Union (EU) has faced many crises in the first quarter of the twenty-first century, and it is important to understand their implications for the integration process. This article investigates how the Covid-19 crisis has impacted public support for European solidarity, which is itself a crucial dimension of the EU polity in the making (Kriesi et al. 2021; Oana et al. 2023). Moments of emergency and radical uncertainty can act as catalysts for solidarity across national boundaries, bringing European citizens closer together in the search for common solutions. However, as the experience from the last decade shows, crises can diminish fellow feelings, occasioning blame games and antagonism, with the risk of unravelling the social bonds that hold the EU polity together. While European solidarity can take many forms, we focus specifically on cross-national fiscal solidarity, i.e. public support for providing financial help to member states in economic and financial difficulties (Bechtel et al. 2014; Gerhards et al. 2019). This has been a demanding form of risk-sharing in the EU and therefore constitutes a crucial test for understanding whether crises contribute to undermine or, by contrast, reinforce EU integration and its micro-foundations.

Several recent studies on the determinants of EU fiscal solidarity stress the role played by European/national identity and cosmopolitan attitudes as well as utilitarian considerations. However, we know less about whether and how these heuristics shape individuals’ attitudes towards solidarity in ‘times of crisis’ in comparison with ‘normal times’. While scholars agree that crisis times are somehow extraordinary, in survey research the effects of crises tend to be left in the background or are simply inferred from the circumstances that characterize the moment when a single survey was fielded. Comparative designs, which contrast ‘normal times’ with ‘crisis times’ data, are rarely used. Empirical evidence about the change in individuals’ solidaristic attitudes during crises is therefore scarce. Although we cannot rely on a panel dataset that would allow for testing causal claims on how the same respondents change their attitudes in the aftermath of a shock, we compare attitudes towards EU solidarity before and after the Covid-19 pandemic. To do so, we draw on observational data taken from two independent cross-national public opinion surveys that were conducted shortly before and soon after the Covid-19 outbreak, and which include the same questions on EU fiscal solidarity along with several covariates.

The first survey was fielded in summer 2019, at a time of relative calm in EU politics: average gross domestic product growth had been stable for several consecutive years and the economic crisis had reached a symbolic endpoint with Greece exiting the last bailout programme in 2018. The number of asylum seekers arriving at the EU’s borders had returned to pre-2015 levels, while the Brexit negotiations were entering their final stage. Furthermore, there was no EU proposal on the table related to fiscal solidarity that would have been salient in the respondents’ minds. One year later, in the summer of 2020, the situation could not have been more different. As a devastating first wave of the Covid-19 pandemic was subsiding, governments were trying to contain the ensuing social and economic fallout. The initial phase of the pandemic occasioned an almost reflexive reversion to ‘nationalized modes of governance’ (Favell and Recchi 2020, p. 884), including border closures and export bans. An acrimonious debate over financial assistance to the most affected member states followed, and the risk that ‘the health and economic challenges generated by Covid-19 [could] strain the frayed political bonds of the EU beyond repair’ was perceived as real (Fierlbeck 2021, p. 18). Nonetheless, in July 2020 EU leaders were able to agree on a massive recovery plan, the Next Generation EU (NGEU) (Bremer et al. 2021). The second survey we rely on was conducted at that point in time, in summer 2020, meaning that, when answering the question on cross-national fiscal solidarity, respondents had a concrete crisis scenario and, possibly, an actual policy proposal in mind.

We use data from the two surveys mentioned above to empirically test an innovative theoretical framework drawn from Kriesi et al. (2021), which contends that the sociospatial distributions of the problem pressure arising from a given crisis affects citizens’ attitudes towards national and supranational policy responses. We expect that the exogenous nature of the Covid-19 crisis, the severity, and the (at least theoretical) symmetry of its consequences reduced the explanatory power of two traditional heuristics that citizens commonly use to shape their attitudes towards EU solidarity: European/national identification and economic self-interest. Our results show that, while the average public support for European solidarity did not increase from 2019 to 2020 in our sample, there were relevant variations across countries. However, the negative associations between exclusive national identification and economic vulnerability on the one hand and EU solidarity on the other were weakened in the aftermath of the Covid-19 crisis.

The next section reviews the recent literature on the determinants of public support for European solidarity, while the following one presents the ongoing debates about the EU polity and its crises and advances our research hypotheses. We then introduce the data and the methods used in the empirical analysis. The fourth section discusses the results. The final one provides conclusions and elaborates on the implications of our findings.

## Public support for European solidarity: The state of the art

European integration raises the question as to what members of an emerging supranational polity ‘owe’ to each other (Sangiovanni 2013). The EU’s foundational documents explicitly include the expectation of solidarity among its citizens and member states. According to the Lisbon Treaty, the EU ‘shall promote economic, social and territorial cohesion, and solidarity among Member States’ (Treaty on European Union, Art. 3). Appeals to solidarity have become ubiquitous, although various actors interpret the concept differently and often deploy it opportunistically (Grimmel 2021). Likewise, popular feelings of solidarity can vary depending on the issue (White 2003). We focus on *cross-national fiscal solidarity*, defined as the willingness to share economic risks among EU member states and measured through public support for an EU mechanism providing financial help to countries in economic and financial hardships (Bechtel et al. 2014; Gerhards et al. 2019). Individual-level utility and identity considerations have been widely found to act as major determinants of public preferences for European solidarity as the ‘two core approaches of preference formation’ (Vasilopoulou and Talving 2020, p. 921; see also Nicoli 2019).^1^ However, while the analytical distinction between these two foundational motivations is useful, it is not absolute. We therefore concur with Jones (2012, p. 690) in his assessment that ‘interest is inextricably bound up in self’—i.e. that the two are interlinked—but assume that in certain situations one or the other may come to the fore.

It is often argued that ‘identity’ and ‘solidarity’ are closely related. Organized solidarity builds on (purportedly) shared identities, and political backlash against cultural and ethnic diversity has the potential to undermine solidaristic arrangements (Banting and Kymlicka 2017). Historically, national citizenship has served as the basis of institutionalized solidarity (Ferrera 2005), and group closure continues to set the limits of solidarity in contemporary societies (Ferrera and Pellegata 2018). In the EU, national identities are institutionalized and well entrenched, and it is unlikely that a ‘thick’ European identity will replace them in the foreseeable future. However, many EU citizens hold nested identities: national, sub-national, and supranational identifications do not necessarily compete, but can also coexist (Medrano and Gutiérrez 2001). In turn, the more one identifies as (also) European, the more she supports EU integration and its various dimensions (Garry and Tilley 2009; Kuhn and Stoeckel 2014; McLaren 2002), including EU fiscal solidarity (Verhaegen 2018).^2^ Identifying exclusively as a national of one’s member state has the opposite effect. That said, Europeanized identities are especially thin—something that people may ‘slip in and out of’ (Favell 2005, p. 1114). For European identity to become salient in socially and politically important ways, people need to be made aware of their ‘identities’, to think and see themselves as Europeans in a certain situation, and to be mobilized *as* Europeans (Risse 2014). To stress this point, and unlike earlier studies (see, for example, Garry and Tilley 2009; McLaren 2002; Verhaegen 2018), in this article we refer to ‘identification’ (instead of ‘identity’), by which we understand an individual’s particularistic self-understanding that shapes social and political action in a non-instrumental manner (Brubaker and Cooper 2000, p. 9). Therefore, while we do believe that identification with the EU can be at least partly captured through surveys, we nonetheless treat such attitudinal measures critically. In fact, our focus on crises highlights the extent to which the social and political relevance of identification is variable and can adjust to changing circumstances.

In comparison with explanations based on identification, the utilitarian approach gives centre stage to the impact of individual cost–benefit analysis on solidaristic attitudes (Hechter 1988). Solidarity depends on one’s socio-economic situation, resources, and interests (Bechtel et al. 2014; Daniele and Geys 2015; Kuhn and Stoeckel 2014; Verhaegen 2018). Indeed, citizens who perceive helping other individuals or member states as threatening their own economic situation are expected to be less likely to support solidarity (Bechtel et al. 2014; Verhaegen 2018), though individuals’ own economic status has a more limited explanatory power in accounting for their position on European fiscal solidarity than identification. Citizens in a weak economic situation tend to be less likely to support providing financial help to other EU member states because they worry that welfare resources, on which they are more likely to depend, would be diverted from their own country towards less deserving ones (Bechtel et al. 2014; Kleider and Stoeckel 2019; Verhaegen 2018). Regarding the role played by sociotropic considerations and national contexts, the literature provides mixed and still inconclusive results. According to Vasilopoulou and Talving (2020), support for EU financial assistance is lower among citizens living in countries with poor macroeconomic performances, while Kuhn and Stoeckel (2014) and Daniele and Geys (2015) have obtained the opposite result.

In sum, we can rely on a substantial body of work to aid us in our exploration of how the mechanisms that drive solidarity may change in times of crisis—an aspect that has received much less attention in the literature. How do these usual heuristics on which individuals rely to shape their attitudes towards EU fiscal solidarity vary in periods of crisis, as opposed to normal times?

## EU solidarity in times of crisis: Theoretical framework and hypotheses

Our point of departure is that individual solidaristic attitudes are responsive to events (White 2003). Crises are such rarely occurring events or moments of ‘Knightian uncertainty’ in which actors’ perceptions of their own self-interest become problematized (Blyth 2002). Crises are typically characterized not only by a sense of urgency but also by extraordinary salience in the media and political debate (Kriesi et al. 2021). As such, crisis politics and crisis policy-making are bound to attract the public’s attention, whose attitudes and preferences cannot be ignored if solutions are to be legitimate or even viable. While numerous empirical studies examine the opinions of Europeans ‘in times of crisis’, only few make systematic pre- and post-crisis comparisons. Potential crisis effects are therefore implied based on the year in which a given survey was fielded (e.g. Verhaegen 2018). We are not alone in diagnosing this shortcoming. When assessing the expectation advanced by Lahusen and Grasso (2018) that crises challenge solidarity, Wallaschek (2019, pp. 262–263) asks: ‘How can we assume that solidarity is under pressure in times of crisis if there is no ex ante and ex post comparison possible?’.

The few studies that systematically compare ‘crisis times’ and ‘normal times’ leave several open questions, and in fact examine different dependent variables than we do (although they are not completely unrelated to EU solidarity). Looking at the effects of the euro crisis on public support for economic integration, Hobolt and Wratil (2015) find that between two points in time, 2005 and 2013, support for economic integration decreased outside the euro area, but remained stable within it. They also show that the rationales driving people’s opinions changed, with identity-based considerations giving way to utilitarian ones in the aftermath of the crisis. More recently, Stockemer et al. (2020, p. 883) examine the effects of the refugee crisis on attitudes towards the EU, immigration, and the link between the two in three different years, 2012, 2014, and 2016, observing no major change and thus concluding that ‘even under a strong external shock, fundamental political attitudes remain constant’.

A general message from these studies is that crises are not as destabilizing for the EU as one may assume. Yet, the scope of the offered explanations remains unclear. Hobolt and Wratil (2015) propose that an *economic* crisis made people more concerned with the *economic* benefits of EU integration, leading to a decrease in identity-based considerations and an increase in utilitarian thinking. The authors acknowledge the shortcomings of this argument, given the narrow focus on ‘attitudes towards monetary integration, which arguably has clearer economic implications than other forms of integration’, so that ‘it is not certain that [their] findings can be generalized to all types of integration support’ (Ibid., 253). It is unclear whether these conclusions also apply to the Covid-19 crisis, or to crises more generally, and whether they extend to another dimension of EU integration support, i.e. EU fiscal solidarity. While their findings may be more pertinent to the economic fallout that accompanied the pandemic, they are certainly less relevant to its health aspects.

This points to the broader problem of issue essentialism, that is, the supposition that the effects of a policy problem can be (at least in part) derived from the substantive issue at hand.^3^ Following Kriesi et al. (2021), we aim to suggest an alternative approach focusing on the sociospatial distribution of the problem pressure that arises in the wake of a crisis and its (perceived) origins (see also Pierson 2011). As Kriesi et al. (2021, pp. 13–14) explain, the pressures induced by crises differ, first, in the extent to which the member states depend on each other for the response (if interdependence is high, this can produce particularly strong demands for coordination) and, second, in the extent to which they affect all member states uniformly (asymmetry creates a differential burden of adjustment in case risks are not shared). If the incidence of the problem is symmetric and cross-national interdependence is high, this gives rise to a ‘common’ crisis situation. Arguably, publics will be more supportive of EU solidarity in such shared circumstances, as opposed to a situation like that of the euro crisis, whereby financial issues were concentrated in a few peripheral EU countries, whose alleged prior fiscal profligacy was pointed out as the root cause of a deeply asymmetrical crisis.

This brings us to a second major factor driving crisis response, i.e. whether the causes of the crisis are widely perceived as having an endogenous or exogenous origin. In the latter case, we can anticipate majorities supporting cross-national solidarity ‘regardless of gains or losses’ for single individuals or their countries (Kriesi et al. 2021, p. 7), since no one member state can be held responsible for the crisis. The Covid-19 crisis fits this scenario since it was generally experienced as a sudden exogenous shock, which was at first identified as a ‘global health emergency that threatens to affect everyone, everywhere’ (Moreira and Hick 2021, p. 2). As the pandemic spread, the economic repercussions of national lockdowns and the diversity of fiscal and social policy responses enacted by national governments contributed to make the impact of the crisis—in terms of the sum of its health and economic dimensions—considerably more asymmetric than it appeared at first sight (Moreira and Hick 2021). Nonetheless, the ‘crisis-time’ data we employed in the analyses were collected in June 2020 (see next section), immediately after the first wave of the Covid-19, when European citizens had plausibly not yet fully recognized the asymmetry of the consequences of the pandemic. Hence, we expect to find that *public support for EU fiscal solidarity increased in the Covid-19 crisis (H1).*

Did the mechanisms driving public support for EU fiscal solidarity also change before and after the outbreak of the pandemic? We expect to find that *the effect of European/national identification on EU fiscal solidarity weakened in the Covid-19 crisis (H2).* Based on Kriesi et al. (2021), we argue that this response is rooted in the exogenous nature of the Covid-19 outbreak shock and its symmetric and interdependent sociospatial distribution. Taken together, these factors should have decoupled solidarity from identitarian considerations. If it is true that, on the one hand, the emergency caused by the pandemic and national lockdowns triggered ‘rally around the flag’ effects that strengthened trust in national governments (Schraff 2021) and, possibly, national identification, on the other the nature of the Covid-19 crisis should have bolstered solidarity over and above contingent identitarian feelings. In other words, we expect that the Covid-19 crisis made room for social bonding *beyond* ‘identity’, possibly also inducing solidaristic feelings among those who do not generally identify as Europeans. Indeed, public support for the EU is multidimensional and individuals’ positive attitudes in one dimension can coexist with scepticism in others (Boomgaarden et al. 2011). While European identification is considered an indicator of regime support for the EU, preferences for policies strengthening solidarity among EU member states represent an evaluation of the policy dimension of support (Hobolt and De Vries 2016). Therefore, even if citizens express a negative evaluation about the current performance of the EU by identifying themselves as exclusive national citizens, they might support a more proactive policy-making role of the EU in increasing their individual and national well-being in a particularly troubling period.

In terms of the relationship between economic self-interest and European solidarity, recall that individuals who feel economically vulnerable are less likely to support fiscal solidarity with other member states (Bechtel et al. 2014; Kleider and Stoeckel 2019; Verhaegen 2018). Again, we expect to find that in the immediate aftermath of the Covid-19 crisis, given the exogenous nature of the shock and the symmetric and still mostly unpredictable nature of an unforeseen pandemic, the heuristic that relies on utilitarian consideration operated differently than in the Eurozone crisis, to which existing studies refer. The specific characteristics of the Covid-19 crisis may have mitigated the distributional consequences of the crisis, thereby inhibiting the activation of a winner/loser mindset at the level of individuals experiencing a common emergency. Therefore, we anticipate that *the (negative) effect of subjective utility considerations on EU fiscal solidarity weakened in the pandemic (H3)*.

## Data and methods

### Data sources and dependent variable: same question, different circumstances

To investigate whether and how a crisis affects the scope and dynamics of cross-national fiscal solidarity in the EU, we exploit two distinct public opinion surveys that were conducted at different points in time: one before and one during the Covid-19 pandemic. Pre-crisis data are taken from the ‘Reconciling Economic and Social Europe: The Role of Values, Ideas and Politics’ (REScEU) survey, which was administered by IPSOS through a computer-assisted web interviewing methodology between 28 June and 2 August 2019 in 10 European countries (Donati et al. 2021). The REScEU survey thus provides a snapshot of a ‘normal time’ when no crisis was in sight and the economic recovery from the Great Recession was well underway. The second survey was fielded in the aftermath of the first wave of Covid-19 in Europe, when the economic and health consequences of the pandemic and of (diversified) national lockdowns were clearly visible to respondents. To wit, we rely on ‘crisis times’ data from the a survey, conducted online by YouGov in seven countries in June 2020, within the framework of the ‘Policy Crisis and Crisis Politics: Sovereignty, Solidarity and Identity in the EU Post-2008' (SOLID) research project. The empirical analyses that follow are performed on the six countries that are present in both surveys: France, Germany, Italy, Spain, Sweden, and the Netherlands. By pooling the two cross-sectional datasets, we obtain a total of 15,461 observations. Table A1 in the Online Appendix provides the descriptive statistics from the pooled sample.

Both surveys employed in our analysis were conducted on national samples stratified by gender, age (18–34, 35–54, 55+), education (lower secondary or less, upper secondary, tertiary), and macro-area of residence (NUTS-1). However, given that these are two separate surveys conducted by different polling companies we compared the structure of the two sub-samples that compose our dataset. Overall, the two sub-samples are representative of the national populations, and we do not detect significant differences in the distribution of the stratification variables that might affect the empirical analyses (see Table A2 in the Online Appendix). On average, the observations collected in 2020 are slightly skewed towards older respondents (55+) at the expense of the 35–54 cohort. While the 2020 sub-sample has only a slightly lower share of respondents with a university degree than in 2019, the difference in the age structure between the two sub-samples is chiefly reflected in the respondents’ occupational status. In the 2020 sub-sample, we observe a higher number of respondents who are out of work (mainly pensioners) compared to the 2019 sub-sample. This difference provides further justification for our choice of using subjective income as an indicator of individual self-interest, as explained in the next section. Furthermore, there are not any notable variations in the distribution of both the dependent variable and the two main covariates, or with respect to the very low number of non-responses. Therefore, the empirical results are unlikely to be driven by a different distribution of respondents on identification and subjective economic perceptions across the two surveys (see Figs. A1, A2, A3, A4 and A5 in the Online Appendix).

The same question on public support for cross-national fiscal solidarity in the EU—the dependent variable in our analysis—was asked in both 2019 and 2020. The wording of the question is the following:

To what extent do you agree or disagree with the following statement: all EU member states, including [COUNTRY], should contribute to a common EU fund to help any other member state facing potential severe economic and financial difficulties in times of crisis.

The response categories consist of a 4-point scale ranging from ‘completely agree’ to ‘completely disagree’. We have recoded the responses into a binary variable coded 1 for respondents who (completely or somewhat) agree and 0 otherwise. This survey item directly taps into fiscal solidarity towards countries ‘facing potential severe economic and financial difficulties in times of crisis’. The reference to the condition of crisis is a standard formulation used, for example, in Eurobarometer waves conducted in 2010 and 2011, in the 2014 European Election Study and in the Transnational European Solidarity Survey (Gerhards et al. 2019). It is meant to conjure up a hypothetical scenario, presumably because a state of crisis is thought to enhance deservingness, which is a key component of how people evaluate solidarity. Therefore, this wording allows us to test the effect of identification and utility on EU fiscal solidarity framed in connection to a supposed emergency in ‘normal times’, versus a situation widely understood and experienced as a crisis—the Covid-19 pandemic.

This formulation of the solidarity question slightly differs from that included in the surveys mentioned above, in that, instead of referring to providing (financial) help from one EU member state to another, it explicitly mentions a mechanism of cross-country redistribution (‘a common EU fund’). Indeed, in June 2020 the public debate on EU responses to the Covid-19 crisis was centred on the use of existing financial rescue funds (the European Stability Mechanism, which was devised in the years of the euro crisis) and on the negotiation for the adoption of the NGEU recovery fund (de la Porte and Jensen 2021). Hence, although it does not capture specific policy features that have been found to influence public support (Bremer et al. 2021), this formulation falls close to concrete instances of cross-national solidarity that EU citizens had in mind from past and present crises.

### Explanatory and control variables

Our main explanatory variables are identification and economic self-interest. We operationalize identification as a binary variable that equals 1 for respondents who declare themselves as ‘exclusive nationals’ to the question ‘Do you see yourself as…?’ (response category ‘[nationality] only’) and 0 for the others (i.e. those who feel ‘European only’, ‘European and [nationality]’, ‘[nationality] and European’). The response ‘none of above’ is treated as a missing value and excluded from the analysis.^4^ For our second explanatory variable, we use subjective income conditions as a proxy for economic self-interest. The variable, labelled ‘low-income condition’, is coded as a dummy that equals 1 for those who find it (very) difficult on their present income and 0 for those who are doing well/coping on their present income. Additional analyses employing the original survey items confirm that dichotomization is the more parsimonious operationalization in the case of both explanatory variables (Online Appendix: Figs. A6, A7, A8 and A9).

We also use several control variables. We consider sociodemographic characteristics: gender (1 = female), age, education, and labour market position (distinguishing between employees with permanent contracts, as well as those with temporary or atypical employment relations, in addition to the self-employed and unemployed, with a residual category including inactive respondents). We also account for respondents’ political dispositions through a binary variable measuring ‘interest in politics’ and a categorical variable on ideological leanings—distinguishing between left (0–4), centre (5), right (6–10), and a residual category for those who did not locate themselves on the 0–10 self-placement scale.

We check the robustness of our empirical results by including in the regression models two further variables that may alter citizens’ attitudes towards European solidarity and their association with our independent variables. The first is ‘trust in national government’, measured through the standardized transformation of a 0–10 scale, where 0 means ‘do not trust at all’ and 10 means ‘trust completely’. This variable is a proxy of the ‘rally around the flag’ effect that several studies have detected during the first phase of the Covid-19 pandemic (Schraff 2021), and accounts for the fact that the extent to which citizens trust their national government can plausibly affect both how citizens identify with the EU and their nation and their willingness to share resources with other member states. The second variable is a proxy for sociotropic economic self-interest that captures respondents’ concern for the state of national economy, and which has been linked to EU solidarity (Daniele and Geys 2015; Kuhn and Stoeckel 2014; Vasilopoulou and Talving 2020). We exclude this variable from the main regression models because the two surveys employed include two different survey items tapping this attitude. The question from the REScEU survey asked whether respondents believed that the national economy had worsened over the five years before the survey entered the field, while in the SOLID survey on Covid-19 the item asked ‘how much of a threat, if any, [was] the Coronavirus outbreak for the national economy’. We have recoded the resulting variable as a dummy taking value 1 for those who thought that the national economy had worsened (or considered the pandemic as a threat to it) and 0 otherwise. Considering these differences between the 2019 and 2020 survey items, the results of the latter robustness checks (see Online Appendix) should be viewed with caution.

### Model specification

Given the binary nature of the dependent variable, we have employed logistic regressions to perform our empirical analyses. We have applied four regression models. The first model regresses public support for European fiscal solidarity on European identification, subjective income conditions and all the control variables described above in the sub-sample, including observations collected in 2019. The second model presents the same specification but includes only observations collected in 2020. The third model is run in the pooled sample and interacts the dummy variable ‘year’ (coded 1 for 2020 and 0 for 2019) with exclusive national identification and low-income condition, respectively. We account for cross-country heterogeneity by including country dummies in the regression models. Finally, we also perform a three-way interaction (identification [income]*year*country) model to test our expectations across countries. We use robust standard errors and post-stratification weights in all models. Given the nonlinear nature of logistic regression models, in the next section we present and discuss graphs displaying the average marginal effects of all the covariates and the interactions on the dependent variable. The regression results are reported in Table A3 (Online Appendix).

## Results

**Fig. 1:**
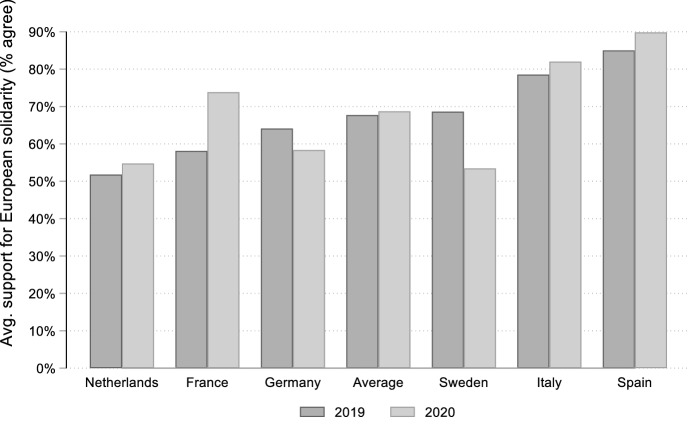
Public support for European fiscal solidarity in 2019 and 2020

In our sample, the average support for EU fiscal solidarity increased slightly between 2019 and 2020. As displayed in Fig. 1, however, this change was trivial (around 1 per cent) and not statistically significant. This result runs against H1, which postulates a rise in public support for European solidarity during the Covid-19 crisis. On a general level, therefore, public attitudes indeed appear stable, in a similar manner to the findings of Stockemer et al. (2020). Looking at the results by country, the average levels of support slightly increase almost everywhere, and quite markedly in France. We detect a minor decrease in Germany and a sizeable one in Sweden. Concomitantly, there are also some differences across sample countries in both 2019 and 2020. This variation mostly ranges along the north–south divide that characterized political conflict in Europe during the euro crisis (Nicoli 2019). This suggests that relevant cross-country variation is possibly driven by citizens’ perceptions of problem pressure for their own country. Citizens became more likely to support European solidarity in those countries that were hit earlier and more significantly by the Covid-19 crisis and, at least for Italy and Spain, which already presented poor macroeconomic performances and high levels of public debt before the crisis. Another noteworthy result is that while respondents chose the original response categories ‘somewhat disagree’ and ‘completely disagree’ in a virtually identical way in the two surveys, in 2020 there was a sizeable expansion in the ‘completely agree’ option and a relative drop in the ‘somewhat agree’ option in comparison with 2019. In other words, while support for EU fiscal solidarity did not change on average or at a general level, it nonetheless became more enthusiastic (see Fig. A1 in Appendix).

**Fig. 2:**
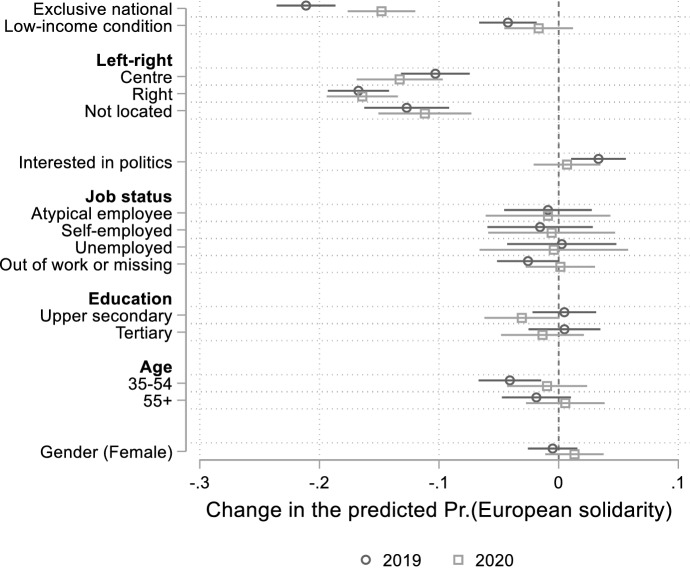
Average marginal effects of covariates on European solidarity in 2019 and 2020

We now discuss empirical results related to H2 and H3, which postulate that the characteristics of the Covid-19 crisis moderate how European identification and subjective economic interest shape citizens’ attitudes towards EU fiscal solidarity. Figs. A10 and A11 in the Online Appendix show the changes in EU solidarity between 2019 and 2020 based on these two explanatory variables. Figure 2 shows the average marginal effects of the independent variables and controls on public support for European solidarity in 2019 and 2020, respectively, with 95 per cent confidence intervals (CIs) reported.

In line with the existing literature, we find that individuals who identify exclusively as ‘nationals’ are significantly less likely to support financial help to EU member states in crisis than those who identify (also) as ‘Europeans’. This negative association is strong and holds in both 2019 and 2020. More importantly, in agreement with H2, in 2020 the magnitude of the association that links exclusive national identification and European solidarity is more than 6 percentage points weaker compared to 2019. The exogenous origin of the Covid-19 crisis, its potentially symmetric impact, and the urgency of a common policy response weakened the scepticism of those strongly identifying with their nation in supporting an EU fund that provides financial help to troubled member states.

Similarly, in 2019 individuals living in low-income condition were less likely to support cross-national fiscal solidarity than those who did not, though the size of this effect (4 percentage points) is much lower than that of ‘exclusive nationals’ (21 percentage points). However, in 2020 subjective income was no more significantly associated with public support for EU fiscal solidarity. This confirms H3 and indicates that identitarian considerations did not give way to utilitarian ones in the aftermath of the Covid-19 crisis.

**Fig. 3:**
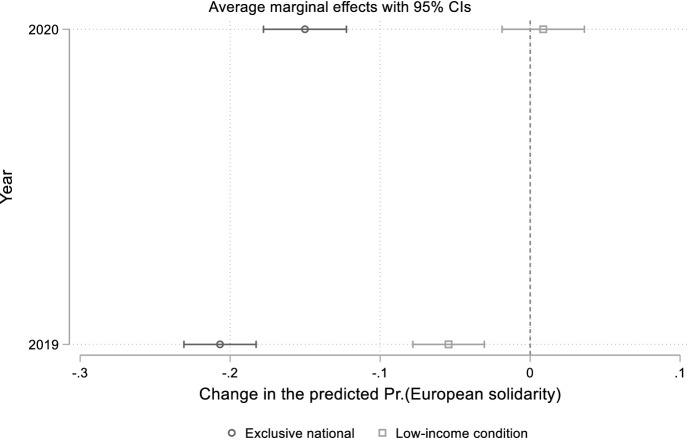
Average marginal effects of exclusive national identification and low-income condition interacted with year on European solidarity

In a third regression model run on the pooled sample we interact exclusive national identification and low-income condition, respectively, with the year (2020) dummy. Figure 3 plots the marginal effects of our two independent variables on the predicted probability of supporting cross-national financial help in 2019 and 2020 with 95 per cent CIs.

Figure 3 confirms the results previously obtained from separate regressions conducted using the 2019 and 2020 sub-samples, respectively. In the aftermath of the Covid-19 crisis, the linkage of European solidarity with both exclusive national identification and low-income condition diminished in comparison with ‘normal times’. Moreover, we again find that in 2020 the association between solidarity and economic self-interest failed to reach levels of statistical significance.

Turning to the effect of control variables on public support for European solidarity, compared to individuals with left-wing ideological leanings, those who locate themselves in the centre or in the right extreme of the ideological axis are less likely to support financial help to other EU member states in crisis, in addition to respondents who refused to express their ideological orientations (around 18 per cent of the total sample). An interest in politics slightly increased the likelihood of supporting European solidarity only in 2019, while during the Covid-19 crisis this association turned out to not be statistically significant. The respondents’ sociodemographic characteristics were instead never significantly associated with public support for European solidarity.

Robustness checks (reported in the Online Appendix) lend further support to our arguments. With the inclusion of sociotropic economic concerns and trust in national government, the main results do not change substantively (Figs. A12 and A13). The negative association linking exclusive nationals and support for European solidarity diminished after the outbreak of the Covid-19 crisis. Concerning subjective economic interest, while in 2019 respondents in low-income condition were slightly less likely to support cross-national financial help than their counterparts, post-Covid-19 crisis they tended to be slightly more supportive of solidarity. Furthermore, to prove that the weakening of the associations linking support for EU fiscal solidarity with identification and subjective economic interest can be explained by the peculiar characteristics of the Covid-19 crisis and not by omitted unobserved factors, we have run a placebo test by assigning each respondent randomly to a dummy variable coded 1 for the treatment group and 0 for the control group. We have then interacted our independent variables with this dummy instead of ‘year’. As expected, we have detected no significant differences in the explanatory power of the independent variables when using this randomized dummy variable in the analysis as a placebo test for ‘year’ (Fig. A14).

**Fig. 4:**
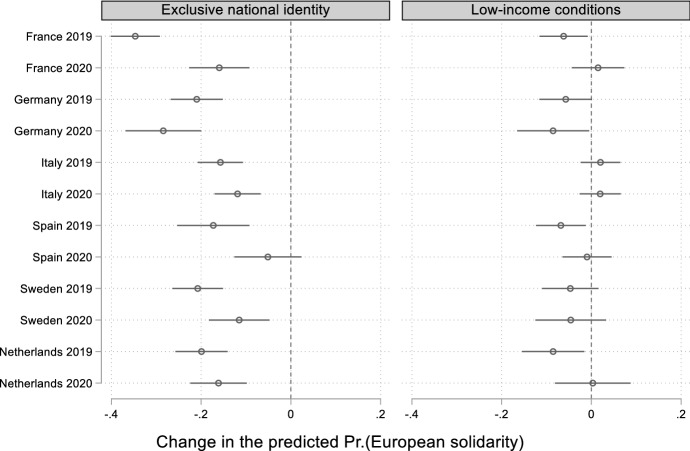
Average marginal effects of exclusive national identification and low-income condition interacted with year and country on European solidarity

Finally, how much national-level variation do our pooled analyses conceal? To answer this question, we compute marginal effects from two three-way interactions between identification/income, year, and country.^5^ Overall, these results reveal a more complex scenario than what we have found in the pooled dataset, but they nonetheless support our core assumptions (Fig. 4; patterns are consistent with those obtained by running separate models in each sample country: see Fig. A15 in the Online Appendix). The effect of national identification diminished across all countries but one, and the direction of change is the same almost everywhere, though most apparent (i.e. statistically significant) in France and Spain. Utilitarian motivations were weaker than identitarian ones in 2019, and in the Covid-19 crisis, they became virtually irrelevant in explaining individual preferences for EU fiscal solidarity. The only exception to this pattern among the countries included in our sample is Germany, where national identification (slightly) increased its negative impact on fiscal solidarity in 2020. This may be due to the move towards EU fiscal solidarity that the German government performed in the negotiations for the NGEU plan. While in the decade of the euro crisis German leaders had vocally opposed risk-pooling among member states, during the pandemic the Merkel-led government changed its position, paving the way for the NGEU agreement. In the short term, German public opinion may have reacted negatively to this a political shift.

## Discussion and conclusions

Crises pose risks that can unravel the social bonds holding the EU polity together, but they can also serve as opportunities for strengthening these bonds. In this article, we have investigated whether and how the Covid-19 crisis moderated public support for cross-national fiscal solidarity in the EU. Whereas in our sample the average level of public attitudes towards European fiscal solidarity in ‘times of crisis’ did not change significantly in comparison with ‘normal times’, we observed notable cross-national variations, with citizens in countries that were hit earlier and more heavily by the Covid-19 crisis becoming more likely to support helping EU member states in crisis. In terms of the structure of attitudes, we found that during the first wave of the pandemic, the two major sources of preference formation, identification and self-interest, played a weaker role in shaping public support for European solidarity. In 2020, ‘exclusive national’ identification remained negatively linked to EU fiscal solidarity, but much less so than in 2019. However, being in a self-reported low-income situation was negatively linked to EU fiscal solidarity only in 2019, but not in 2020. We argue that the increase in solidarity among exclusive nationals and ‘self-interested’ individuals can be explained by the impact of the pandemic as a ‘common crisis’, which affected all member states in a symmetric manner and induced interdependencies among them (cf. Bremer et al. 2021; Kriesi et al. 2021). These findings echo those of Kriesi and Bojár (2023), who compare policy responses to the refugee and Covid-19 crises and argue that successful reform was possible in the case of the latter but not the former because of the original problem pressure and the different distribution of spatial incidence in the two crises. More generally, we want to underline that individual crises may have varying effects, which do not necessarily entail destructive implications for the EU polity.

Our analyses confirm the idea that identification plays an important role in developing pan-European solidarity: only the identitarian source remains relevant in 2020, whereas self-interest motivations falter. While not assuming a rigid boundary between the two main drivers of behaviour, we can state that the Covid-19 crisis created a situation of uncertainty in which it was unclear what one’s interests were, thus bringing the identitarian heuristic to the fore. At the same time, our results show that solidarity and identification are not as firmly linked as is sometimes assumed: exclusive nationals were more prone to show solidarity in the aftermath of the first wave of Covid-19 than in 2019. This has important implications for the ongoing debate on the lack of a pan-European identity as an impediment for EU integration (for a thoughtful discussion of what to [not] expect from an EU identity, see: Cram 2012). Robust forms of solidarity can also find support among those declaring themselves to be ‘exclusive nationals’, at least in a ‘common’ crisis. In other words, solidarity and identification are to some extent alternative modes of social bonding. This supports the conclusions of Jones (2012, p. 699), namely that ‘Europeans do not need a common identity to promote European integration and common identity formation should not be viewed as a measure of European integration’s success’. Our findings show that, even among those with a sheer lack of European identification, anti-solidaristic attitudes can be mitigated when a crisis has given characteristics (or when it is framed in a given way). Certainly, the agency of political actors also matters in shaping the way in which a crisis is perceived by and affects public opinion. We leave this aspect to further research on party cues and political leaders’ discursive strategies.

Our paper also comes with some limitations which could be tackled in future research. While our use of pooled cross sections before and after a relevant crisis is a step towards a more sophisticated understanding of crisis effects, only panel data would allow for more robust conclusions on how public attitudes towards European solidarity do change in the aftermath of a crisis. A further open question is whether crisis effects return to where they were before the crisis or whether they are more durable. Russo (2023) detect changes in key public attitudes during the pandemic, i.e. between 2020 and 2021. Conversely, Stockemer et al. (2020) compare integration and migration-related attitudes before, during, and after the refugee crisis and find them to be remarkably stable. Lastly, with only six countries included in our two surveys, it is difficult to test the impact of macro-level variables that might play a role in shaping public support for EU fiscal solidarity (Lahusen 2020, 16 ff., Vasilopoulou and Talving 2020). Future studies should address these weaknesses to provide a more complete understanding of how different crises shape public support for European solidarity.

## Supplementary Information

Below is the link to the electronic supplementary material.

Supplementary file1 (DOCX 820 KB)

## Footnotes

1 A third strand of research focuses on the role of general political orientations and party cues as important heuristics that citizens use to shape their opinions (Bechtel et al. 2014; Daniele and Geys 2015; Kleider and Stoeckel 2019).

2 Vasilopoulou and Talving (2020) nonetheless warn that general preferences for EU integration are not coterminous with preferences related to fiscal solidarity: the two should not be conflated.

3 Greer and Löblová (2017) make a similar observation in a different context.

4 To check the robustness of our results, we have also rerun the empirical analyses by using a different operationalization of the European identification variable that includes the original ‘none of the above’ category among those who ‘feel (also) as Europeans’. The main results do not change (see Appendix).

5 The results are very similar when we run separate models with interactions between the year dummy and identification and income, respectively, in single sample countries (see Online Appendix).
