# Supplementary material for: Closer in hard times? The drivers of European solidarity in ‘normal’ and ‘crisis’ times
Source: Comp Eur Polit. 2023 Feb 7:1–19. Online ahead of print. doi: 10.1057/s41295-023-00332-w (PMC9902254; doi:10.1057/s41295-023-00332-w)
Supplement: Supplementary file 1 — Supplementary file1 (DOCX 820 KB) [file 41295_2023_332_MOESM1_ESM.docx]

**Closer in hard times? The drivers of European solidarity in ‘normal’ and ‘crisis’ times**

**Online appendix**

Table A1**.** Descriptive statistics, pooled sample

| Variable | Obs. | Mean/percentage | Std. Dev. | Min | Max |
| --- | --- | --- | --- | --- | --- |
| Support for European fiscal solidarity | 14207 | .681 |  | 0 | 1 |
| Exclusive national identity | 14048 | .322 |  | 0 | 1 |
| Low-income condition | 14998 | .299 |  | 0 | 1 |
| **Left-right self-placement** |  |  |  |  |  |
| Left | 15461 | .316 |  | 0 | 1 |
| Centre | 15461 | .181 |  | 0 | 1 |
| Right | 15461 | .324 |  | 0 | 1 |
| Not located | 15461 | .179 |  | 0 | 1 |
| Interested in politics | 15119 | .619 |  | 0 | 1 |
| **Job status** |  |  |  |  |  |
| Permanent employee | 15461 | .408 |  | 0 | 1 |
| Atypical employee | 15461 | .09 |  | 0 | 1 |
| Self-employed | 15461 | .059 |  | 0 | 1 |
| Unemployed | 15461 | .062 |  | 0 | 1 |
| Out of work | 15461 | .381 |  | 0 | 1 |
| **Education** |  |  |  |  |  |
| Lower secondary or less | 15438 | .268 |  | 0 | 1 |
| Upper secondary | 15438 | .421 |  | 0 | 1 |
| Tertiary | 15438 | .311 |  | 0 | 1 |
| **Age class** |  |  |  |  |  |
| 18-34 | 15461 | .249 |  | 0 | 1 |
| 35-54 | 15461 | .398 |  | 0 | 1 |
| 55+ | 15461 | .353 |  | 0 | 1 |
| Gender (Female) | 15461 | .507 |  | 0 | 1 |
| Exclusive national identity (2) | 15052 | .3 |  | 0 | 1 |
| Sociotropic economic concerns | 14960 | .595 |  | 0 | 1 |
| Std. trust in national government | 15090 | 0 | 1 | -1.608 | 1.935 |
| Note: for categorical and dummy variables entries in the third column are percentages while for continuous variables are means.  Source: 2019 REScEU survey and 2020 SOLID survey | | | | | |

Table A2. Structure of the 2019 and 2020 sub-samples

|  | 2019 | | 2020 | |
| --- | --- | --- | --- | --- |
|  | Obs. | Perc. | Obs. | Perc. |
| European fiscal solidarity | 8341 | .677 | 5866 | .688 |
| European identification | 8362 | .329 | 5686 | .312 |
| Low-income condition | 8855 | .292 | 6143 | .309 |
| **Left-right self-placement** |  |  |  |  |
| Left | 9051 | .321 | 6410 | .309 |
| Centre | 9051 | .189 | 6410 | .169 |
| Right | 9051 | .329 | 6410 | .317 |
| Not located | 9051 | .161 | 6410 | .206 |
| Interest in politics | 8879 | .617 | 6240 | .622 |
| **Job status** |  |  |  |  |
| Permanent employee | 9051 | .429 | 6410 | .379 |
| Atypical employee | 9051 | .104 | 6410 | .07 |
| Self-employed | 9051 | .06 | 6410 | .057 |
| Unemployed | 9051 | .068 | 6410 | .052 |
| Out of work | 9051 | .339 | 6410 | .441 |
| **Education** |  |  |  |  |
| Lower secondary or less | 9051 | .258 | 6387 | .282 |
| Upper secondary | 9051 | .422 | 6387 | .42 |
| Tertiary | 9051 | .32 | 6387 | .298 |
| **Age class** |  |  |  |  |
| 18-34 | 9051 | .256 | 6410 | .238 |
| 35-54 | 9051 | .433 | 6410 | .35 |
| 55+ | 9051 | .311 | 6410 | .412 |
| Gender | 9051 | .504 | 6410 | .51 |
| Exclusive national identity (2) | 8893 | .309 | 6159 | .288 |
| Sociotropic economic concerns | 8550 | .4 | 6410 | .855 |

Source: 2019 REScEU survey and 2020 SOLID survey

Figure A1. Distribution of the original survey item measuring EU fiscal solidarity

Source: 2019 REScEU survey and 2020 SOLID survey

Figure A2. Distribution of the original survey item measuring identification

Source: 2019 REScEU survey and 2020 SOLID survey

Figure A3. Distribution of the original survey item measuring subjective income

Source: 2019 REScEU survey and 2020 SOLID survey

Comments on Figs. A1, A2 and A3: We have compared the original survey items on EU fiscal solidarity, identification and subjective income to understand whether the recoding of these variables covers some differences between the two sub-samples. For all the three items the share of ‘Don’t know’ is lower than 10%. The share of ‘Don’t know’ for solidarity is about the same in 2019 and 2020 (Fig. A1), while the share of ‘Don’t know’ for identification and subjective income increased in 2020, compared to 2019 (Figs. A2 and A3). However, for both items it remains very low (around 4%). As regards EU fiscal solidarity, in 2020 the percentage of ‘completely agree’ increased and the percentage of somewhat agree decreased compared to 2019. We therefore can dichotomize this variable (it actually makes the shares even more consistent across the samples). Regarding identification (Fig. A2), the percentage of ‘nationality only’ and ‘nationality and European’ decreased in 2020 and the percentage of European only increased slightly. However, in both 2019 and 2020 ‘European only’ and ‘European and national’ are residual categories confirming the choice to dichotomize the variable into ‘Exclusive nationals’ and ‘Feel (also) European’. Regarding subjective income, there are no big differences between the 2019 and 2020 sub-samples (Fig. A3).

Figure A4. Distribution of the recoded identification variable across country-years

Source: 2019 REScEU survey and 2020 SOLID survey

Concerning identification (Fig. A4), we see the following: In France and Germany in 2020 the percentage of ‘exclusive nationals’ decreased compared to 2019. In Spain, the Netherlands and Sweden we find no differences between 2019 and 2020. Italy is the only country where the percentage of ‘exclusive nationals’ increased in 2020 compared to 2019.

Figure A5. Distribution of the recoded subjective income variable across country-years

Source: 2019 REScEU survey and 2020 SOLID survey

Concerning subjective income (Fig. A5) we see the following: In France and Italy the share of respondents in low-income conditions increased in 2020 compared to 2019. In Spain and Sweden, the share of respondents in low-income conditions slightly increased in 2020 compared to 2019. In Germany and the Netherlands the share of respondents in low-income conditions slightly decreased in 2020 compared to 2019.

Figure A6. Predicted probability of supporting EU fiscal solidarity (range: 0-1) in 2019 and 2020 by respondents in different categories of identification.

Source: 2019 REScEU survey and 2020 SOLID survey

Self-proclaimed ‘exclusive nationals’ have a significantly lower probability to support EU fiscal solidarity than all the other categories in both 2019 and 2020. On the contrary, there are no statistically significant differences between all the other categories. Furthermore, in 2020, compared to 2019, the predicted probability that ‘exclusive nationals’ support European solidarity is significantly higher, meaning that the association between exclusive national identification and support for European solidarity weakened during the first wave of the pandemic (as in Figure 3 in the Article). Fig. A6 confirms that the main dividing line regarding identification separates those who consider themselves as exclusively national citizens and all the other categories.

Figure A7. Predicted probability of supporting EU fiscal solidarity in 2019 and 2020 by respondents in different income categories

Source: 2019 REScEU survey and 2020 SOLID survey

Fig. A7 shows that, in 2019, those in worse economic conditions tended to be less likely to support EU fiscal solidarity than those in better economic conditions. In 2020, the probability of supporting solidarity for those who find it (very) difficult on present income is higher than in 2019 (statistically significant only for those who find it difficult on present income). Compared to 2019, in 2020 we find the opposite scenario for those who cope or live comfortably on present income, but the changes in the predicted probability are not statistically significant. This variation confirms our choice of dichotomizing subjective income between low-income and high-income respondents.

Figure A8. Average marginal effects of covariates on European solidarity in 2019 and 2020 with a different operationalization of exclusive national identification

Source: 2019 REScEU survey and 2020 SOLID survey

Figure A9. Average marginal effects of European identification and subjective economic interested interacted with year on European solidarity using a different operationalization of exclusive national identification

Source: 2019 REScEU survey and 2020 SOLID survey

Table A3. Regression results, main models

|  | Model 1 | Model 2 | Model 3 | Model 4 | Model 5 |
| --- | --- | --- | --- | --- | --- |
|  | 2019 | 2020 | Pooled  dataset | Pooled dataset | Pooled dataset |
|  |  |  |  |  |  |
| Exclusive national identification | -1.034*** | -0.768*** | -1.015*** | -0.920*** | -1.047*** |
|  | (0.059) | (0.073) | (0.059) | (0.130) | (0.059) |
| Low-income condition | -0.225*** | -0.091 | -0.288*** | -0.234*** | -0.270* |
|  | (0.064) | (0.080) | (0.064) | (0.064) | (0.140) |
| Year(2020) |  |  | -0.157*** | -0.376*** | -0.453*** |
|  |  |  | (0.059) | (0.115) | (0.114) |
| Exclusive national identification*Year(2020) |  |  | 0.268*** | -0.294 | 0.292*** |
|  |  |  | (0.089) | (0.233) | (0.091) |
| Low-income condition*Year(2020) |  |  | 0.335*** | 0.170* | -0.102 |
|  |  |  | (0.095) | (0.099) | (0.226) |
| Country |  |  |  |  |  |
| *Germany (reference category)* |  |  |  |  |  |
| France | 0.222** | 0.886*** | 0.457*** | 0.061 | -0.153 |
|  | (0.088) | (0.111) | (0.068) | (0.110) | (0.104) |
| Italy | 0.347*** | -0.031 | 0.200*** | 0.934*** | 0.775*** |
|  | (0.092) | (0.104) | (0.069) | (0.126) | (0.119) |
| Spain | 1.330*** | 1.519*** | 1.391*** | 0.991*** | 1.010*** |
|  | (0.101) | (0.121) | (0.077) | (0.122) | (0.129) |
| Sweden | 1.338*** | 1.727*** | 1.477*** | 0.430*** | 0.400*** |
|  | (0.108) | (0.139) | (0.084) | (0.121) | (0.110) |
| The Netherlands | 0.779*** | -0.054 | 0.409*** | -0.415*** | -0.353*** |
|  | (0.096) | (0.100) | (0.069) | (0.113) | (0.105) |
| Exclusive national identification*France |  |  |  | -0.595*** |  |
|  |  |  |  | (0.182) |  |
| Exclusive national identification*Italy |  |  |  | -0.034 |  |
|  |  |  |  | (0.196) |  |
| Exclusive national identification*Spain |  |  |  | -0.135 |  |
|  |  |  |  | (0.248) |  |
| Exclusive national identification*Sweden |  |  |  | -0.086 |  |
|  |  |  |  | (0.189) |  |
| Exclusive national identification*Netherlands |  |  |  | 0.080 |  |
|  |  |  |  | (0.182) |  |
| 2020*France |  |  |  | 0.854*** | 0.996*** |
|  |  |  |  | (0.175) | (0.170) |
| 2020*Italy |  |  |  | 0.683*** | 0.674*** |
|  |  |  |  | (0.209) | (0.193) |
| 2020*Spain |  |  |  | 0.685*** | 0.751*** |
|  |  |  |  | (0.197) | (0.211) |
| 2020*Sweden |  |  |  | -0.619*** | -0.427*** |
|  |  |  |  | (0.172) | (0.160) |
| 2020*Netherlands |  |  |  | 0.367** | 0.348** |
|  |  |  |  | (0.166) | (0.153) |
| Exclusive national identification*France*2020 |  |  |  | 0.966*** |  |
|  |  |  |  | (0.317) |  |
| Exclusive national identification*Italy*2020 |  |  |  | 0.346 |  |
|  |  |  |  | (0.336) |  |
| Exclusive national identification*Spain*2020 |  |  |  | 0.878** |  |
|  |  |  |  | (0.444) |  |
| Exclusive national identification*Sweden*2020 |  |  |  | 0.819*** |  |
|  |  |  |  | (0.307) |  |
| Exclusive national identification*Netherlands*2020 |  |  |  | 0.456 |  |
|  |  |  |  | (0.299) |  |
| Low-income condition*France |  |  |  |  | -0.014 |
|  |  |  |  |  | (0.187) |
| Low-income condition*Italy |  |  |  |  | 0.407** |
|  |  |  |  |  | (0.207) |
| Low-income condition*Spain |  |  |  |  | -0.175 |
|  |  |  |  |  | (0.225) |
| Low-income condition*Sweden |  |  |  |  | 0.022 |
|  |  |  |  |  | (0.216) |
| Low-income condition*Netherlands |  |  |  |  | -0.112 |
|  |  |  |  |  | (0.211) |
| Low-income condition*France*2020 |  |  |  |  | 0.472 |
|  |  |  |  |  | (0.309) |
| Low-income condition*Italy*2020 |  |  |  |  | 0.125 |
|  |  |  |  |  | (0.332) |
| Low-income condition*Spain*2020 |  |  |  |  | 0.458 |
|  |  |  |  |  | (0.382) |
| Low-income condition*Sweden*2020 |  |  |  |  | 0.149 |
|  |  |  |  |  | (0.329) |
| Low-income condition*Netherlands*2020 |  |  |  |  | 0.498 |
|  |  |  |  |  | (0.334) |
| Left-right self-placement |  |  |  |  |  |
| *Left (reference category)* |  |  |  |  |  |
| Centre | -0.586*** | -0.760*** | -0.635*** | -0.652*** | -0.654*** |
|  | (0.083) | (0.103) | (0.064) | (0.065) | (0.065) |
| Right | -0.905*** | -0.916*** | -0.899*** | -0.906*** | -0.914*** |
|  | (0.073) | (0.087) | (0.055) | (0.056) | (0.056) |
| Not located | -0.708*** | -0.651*** | -0.659*** | -0.667*** | -0.681*** |
|  | (0.097) | (0.113) | (0.073) | (0.074) | (0.074) |
| Interested in politics | 0.178*** | 0.038 | 0.114** | 0.121** | 0.123** |
|  | (0.062) | (0.078) | (0.048) | (0.049) | (0.049) |
| Job status |  |  |  |  |  |
| *Permanent employee (reference category)* |  |  |  |  |  |
| Atypical employee | -0.048 | -0.049 | -0.022 | -0.036 | -0.035 |
|  | (0.101) | (0.145) | (0.083) | (0.083) | (0.083) |
| Self-employed | -0.084 | -0.032 | -0.055 | -0.069 | -0.064 |
|  | (0.120) | (0.148) | (0.093) | (0.093) | (0.093) |
| Unemployed | 0.014 | -0.022 | -0.016 | 0.005 | 0.005 |
|  | (0.128) | (0.173) | (0.102) | (0.104) | (0.103) |
| Out of work | -0.138* | 0.008 | -0.043 | -0.079 | -0.069 |
|  | (0.071) | (0.081) | (0.053) | (0.054) | (0.054) |
| Education |  |  |  |  |  |
| Lower secondary or less (reference category) |  |  |  |  |  |
| Upper secondary | 0.026 | -0.170* | -0.044 | -0.049 | -0.048 |
|  | (0.073) | (0.088) | (0.056) | (0.056) | (0.056) |
| Tertiary | 0.026 | -0.075 | -0.003 | -0.013 | -0.016 |
|  | (0.082) | (0.099) | (0.063) | (0.063) | (0.063) |
| Age |  |  |  |  |  |
| *18-34 (reference category)* |  |  |  |  |  |
| 35-54 | -0.220*** | -0.053 | -0.162*** | -0.049 | -0.048 |
|  | (0.073) | (0.093) | (0.057) | (0.056) | (0.056) |
| 55+ | -0.102 | 0.031 | -0.074 | -0.013 | -0.016 |
|  | (0.081) | (0.093) | (0.061) | (0.063) | (0.063) |
| gender (female) | -0.027 | 0.073 | 0.006 | 0.014 | 0.007 |
|  | (0.057) | (0.068) | (0.043) | (0.044) | (0.044) |
|  |  |  |  |  |  |
| Constant | 1.201*** | 1.173*** | 1.236*** | 1.551*** | 1.612*** |
|  | (0.132) | (0.159) | (0.105) | (0.117) | (0.116) |
|  |  |  |  |  |  |
| Observations | 7,624 | 5,117 | 12,741 | 12,741 | 12,741 |

Note: Robust standard errors in parentheses; post-stratification weight applied. *** p<0.01, ** p<0.05, * p<0.1

Source: 2019 REScEU survey and 2020 SOLID survey

Figure A10. Changes in public support for EU fiscal solidarity in 2019 and 2020 among exclusive nationals and those who feel (also) European.

Source: 2019 REScEU survey and 2020 SOLID survey

Figure A11. Changes in public support for EU fiscal solidarity in 2019 and 2020 among respondents in good and low-income conditions.

Source: 2019 REScEU survey and 2020 SOLID survey

Figure A12. Average marginal effects of covariates on European solidarity in 2019 and 2020 including sociotropic economic concerns and trust in national government among controls

Source: 2019 REScEU survey and 2020 SOLID survey

Figure A13. Average marginal effects of European identification and subjective economic interested interacted with year on European solidarity, net of sociotropic economic concerns and trust in national government among controls

Source: 2019 REScEU survey and 2020 SOLID survey

Figure A14. Average marginal effects of European identification and subjective economic concerns on European solidarity interacted with a placebo grouping respondents randomly in two groups, before the crisis and after the crisis

Source: 2019 REScEU survey and 2020 SOLID survey

Figure A15. Change in the predicted probabilities of supporting EU fiscal solidarity for respondents identifying as exclusive nationals (compared to those who identify [also] as European) and those with a lower income (compared to those with a higher income) in 2019 and 2020, separate models in single sample countries.

Source: 2019 REScEU survey and 2020 SOLID survey
